# Approaches combining mice and *Drosophila melanogaster *models to decipher human sepsis

**DOI:** 10.1186/cc14070

**Published:** 2014-12-03

**Authors:** R Ramy, S Garnier, L Pradel, L Röder, B Loriod, A Defaye, L Perrin, P Rihet, C Nguyen

**Affiliations:** 1 Aix-Marseille Université, Inserm, UMRS 1090, TAGC, Parc scientifique de Luminy, Marseille, France

## Introduction

Sepsis is a complex and heterogeneous syndrome in which inflammatory and infection mechanisms are implicated. Thus, it is difficult to differentiate those mechanisms in mammals where inflammation is a highly complex biological process. In our laboratory, besides studying sepsis in human blood biological samples, we choose to investigate a murine model in ordered to describe a global transcriptome overview of critical events occurring in the blood, brain and lung during an induced non-infection inflammation. We decided to complete our view with another animal model where the inflammatory process is less complex and mainly achieved through innate immunity *Drosophila melanogaster*.

## Methods

Female C57BL6/J mice received an intravenous oleic acid (OA) injection to induce a controlled inflammation. Lung, brain and peripheral blood mononuclear cell (PBMC) expression patterns were analyzed using an Agilent 60K cDNA mouse microarray. The enrichment of canonical pathways revealed marked changes in pathways involving the immune and inflammatory responses. The inflammatory process in *D. melanogaster *shares common mechanisms in innate immunity with mammals (Toll pathway/TLR). We decided to set up a model, called the double-hit model, where drosophila females aged from 7 to 10 days have been injured with a needle (inflammatory hit) before being infected with a needle that has been dipped in a bacterial solution of *Pseudomonas entomophila *(infectious hit). The next step will be to investigate the transcriptome in order to highlight the differences and have a global view of biological mechanisms involved; and also look for genetic factors involved in this experiment. The DGRP (Drosophila Genomic Reference Panel) project is a panel of 200 inbred fly lines that have been fully sequenced.

## Results

Strikingly, in mice models, all significant pathways identified in the brain were also significant in the lung. The inflammatory responses oscillated between proinflammatory and anti-inflammatory response in both the lung and brain, the time course, however, being different in the two organs. In PBMC, we observed a significant response delay after OA injection and the pathways identified differed from those identified in the lung and brain. Our second objective will be to use this model of the expression to set up a tool to analyze precisely the effect of an infection in a second hit during the sterile inflammation activation/repression time courses. In *D. melanogaster*, we recorded their survival in comparison with flies that only received the infectious hit and observed a difference of ~40%. Flies that have received a non-infectious double-hit showed a 10% decrease in survival rate. We will perform the double-hit experiment with all the lines of the DGRP and then through genome-wide association study. We expected to identify single nucleotide polymorphisms that are associated with resistance or susceptibility to the double-hit.

## Conclusion

In mice, we assessed gene expression profiles in mouse lung, brain and PBMC associated with a lung inflammation during a 24-hour time course. Overall, our microarray analysis provides a global and detailed overview of critical transcriptional events occurring in the blood, brain and lung. The analysis of gene functional annotation revealed several major features. First, many genes were upregulated or downregulated over the time in the lung, brain, and blood. Second, the analysis revealed pathways clearly related to inflammation mainly in the lung and brain. The strong inflammation observed in the brain, and a limited inflammation in the blood, indicates that blood gene expression profiles poorly reflect those observed in the lung and brain. Third, all the pathways identified in the brain were also identified in the lung, although a few common genes were characterized; it should also be stressed that there was a delay response in the brain.

